# Supplementary material for: Investigating the relation between self-assessment and patients’ assessments of physicians-in-training empathy: a multicentric, observational, cross-sectional study in three teaching hospitals in Brazil
Source: BMJ Open. 2019 Jun 25;9(6):e029356. doi: 10.1136/bmjopen-2019-029356 (PMC6597646; doi:10.1136/bmjopen-2019-029356)
Supplement: Author's manuscript [file bmjopen-2019-029356.draft_revisions.pdf]

BMJ Open is committed to open peer review. As part of this commitment we make the peer review history of every article we publish publicly available.

When an article is published we post the peer reviewers' comments and the authors' responses online. We also post the versions of the paper that were used during peer review. These are the versions that the peer review comments apply to.

The versions of the paper that follow are the versions that were submitted during the peer review process. They are not the versions of record or the final published versions. They should not be cited or distributed as the published version of this manuscript.

BMJ Open is an open access journal and the full, final, typeset and author-corrected version of record of the manuscript is available on our site with no access controls, subscription charges or pay-per-view fees (http://bmjopen.bmj.com).

If you have any questions on BMJ Open's open peer review process please email info.bmjopen@bmj.com

BMJ Open

Investigating the relation between self-assessment of empathy by physicians in training with patients' assessments after clinical encounters: a multicentric observational cross-sectional study

Journal:	*BMJ Open*
Manuscript ID	bmjopen-2019-029356
Article Type:	Research
Date Submitted by the Author:	22-Jan-2019

Complete List of Authors:	Bernardo, Mônica; Pontificia Universidade Catolica de Sao Paulo Faculdade de Ciencias Medicas e da Saude, Radiology<br>Cecilio-Fernandes, Dario; University Medical Center Groningen, CEDAR - Center for Education Development and Research in Health Professions<br>Lima, Alba; Medical Faculty of São Jose do Rio Preto<br>Silva, Julian; Universidade Estadual de Campinas Faculdade de Ciencias Medicas, Internal Medicine<br>Ceccato, Hugo; Universidade Estadual de Campinas Faculdade de Ciencias Medicas, Internal Medicine<br>Costa, Manuel; School of Health Sciences, University of Minho, Life and Health Sciences Research Institute<br>de Carvalho-Filho, Marco Antonio; University Medical Center Groningen, CEDAR - Center for Educational Development and Research in Health Sciences; Universidade Estadual de Campinas Faculdade de Ciencias Medicas, Internal Medicine

Keywords:	Empathy, MEDICAL EDUCATION & TRAINING, Internship, Residency

SCHOLARONE™
Manuscripts

Title: Investigating the relation between self-assessment of empathy by physicians in training with patients' assessments after clinical encounters: a multicentric observational cross-sectional study.

Mônica O. Bernardo, MD*, Dario Cecilio-Fernandes*, PhD, Alba R. A. Lima, PhD, Julian F. Silva, Hugo D. Ceccato, Manuel J. Costa, PhD, Marco A. Carvalho Filho, MD, PhD.

*These authors contributed equally to this work.

Authors' contributions:

MOL, MJC and MACF have substantially contributed to the design of the work. MOL, ARAL, JFS and HDC were responsible for the acquisition of data. DCF was responsible for the data analyses. DCF, MJC and MACF were responsible for the interpretation of the data. MOL and DCF were responsible for the first draft of the paper. All the authors have critically revised and approved the final version of the paper. All authors are accountable for all the aspects of the work.

Mônica Oliveira Bernardo* is PhD candidate in Medical Education, School of Medical Sciences, University of Campinas, Campinas, São Paul, Brazil, and assistant professor of medicine, Radiology Department, Faculty of Medicine, Catholic University of São Paulo, Sorocaba, São Paulo, Brazil.

For peer review only

Dario Cecilio-Fernandes* is Researcher at the Center for Education Development and Research in Health Professions (CEDAR), research group LEARN, the University of Groningen, University Medical Center Groningen; ORCID: http://orcid.org/0000-0002-8746-1680

Alba Regina de Abreu Lima is professor of medicine, Medical Faculty of São Jose do Rio Preto, São Jose do Rio Preto, São Paulo, Brazil.

Julian Furtado Silva is a senior medical student, School of Medical Sciences, University of Campinas, Campinas, São Paulo, Brazil.

Hugo Dugolin Ceccato, is a senior medical student, School of Medical Sciences, University of Campinas, Campinas, São Paulo, Brazil.

Manuel João Costa, pro-rector, professor of medicine, Life and Health Sciences Research Institute, School of Health Sciences, University of Minho, Braga, Portugal

Marco Antonio de Carvalho-Filho is professor of medicine, undergraduate and postgraduate courses, Emergency Department, School of Medical Sciences, University of Campinas, Campinas, São Paulo, Brazil, Center for Educational Development Innovation and Research, University Medical Center, University of Groningen, Groningen; ORCID: http://orcid.org/0000-0001-7008-4092

For peer review only

Correspondence should be addressed to Marco Antonio de Carvalho-Filho, Onderwijsinstituut (OWI), UMCG, A Deusinglaan 1 (FC40), Building 3219, 9713 AV Groningen, The Netherlands, +31621330898 macarvalhofilho@gmail.com, m.a.de.carvalho.filho@umcg.nl.

Number all words: 2.522

For peer review only

ABSTRACT

Objectives: This study investigated the relation between senior medical students and residents self-assessed empathy levels with empathy levels as perceived by their patients in real clinical encounters. The authors also examined whether patients' measures were valid and reliable as assessment methods for senior medical students and residents.

Design: A multicentric observational cross-sectional study

Setting: This study was conducted in three public teaching hospitals.

Participants: From the 668 patients invited to participate in this research, 566 (84,7%) agreed. Of those, 238 (42%) were male, and 328 (58%) female patients. From the invited 112 physicians in training, 86 (76.8%) agreed. Of the 86 physicians in training, 35 (41%) were final years medical students and 51 (59%) were residents from clinical and surgical specialties. The gender distribution was 39 (45%) males and 47 (51%) females.

Primary and secondary outcome measures: Physicians in training filled the Jefferson Scale of Physician Empathy (JSE) and the Interpersonal Reactivity Index (IRI). The patients answered the Jefferson Scale of Patient's Perceptions of Physician Empathy (JSPPPE) and the Consultation and Relational Empathy Scale (CARE).

Results: There was no significant correlation between patients' measures with students and residents self-assessment, except for a weak correlation (0,241, $p<0.01$) between the JSPPPE score and the JSE Compassionate Care sub-score. Medical students demonstrated higher levels of empathy than residents in both self-assessment and patients' measures. CARE and JSPPPE scales proved to be valid and reliable instruments.

Conclusions: The findings reinforced the need to add the patient's perspectives in the evaluation of educational interventions to foster empathy. Adding patients' perspectives may also send the

For peer review only

message that a good doctor should acknowledge, reflect on, and react to patients' opinions and views.

Strengths and limitations of this study:

- This was a multicentric study involving three public teaching hospitals.
- The higher ratio of patients per doctors allows to decrease the bias and obtain a reliable measurement of the level of empathy of the physician in training.
- Elements, like the time spent in the consultation and/or waiting room, that could influence patients' perspective were not explored.
- Multiple perspectives of the level of empathy of the physicians in training were taken.

Key words: Empathy. Education, Medical. Internship and Residency. Patients.

For peer review only

INTRODUCTION

Physician empathy is crucial for patient care, impacting patients' satisfaction and outcomes.[1 2] Empathy enhances professional fulfillment,[1] diminishes physicians' burnout,[3] and contributes to medical students' clinical competence.[4] Based on the relevance of empathy to medical practice, undergraduate medical curricula should provide learning opportunities to nurture student empathy towards patients.[4] Nevertheless, research data is controversial on whether the current medical training has a negative impact on empathy levels of students (for a discussion see Ferreira-Valente et al.,[5]). The majority of findings in those studies relies exclusively on self-assessment instruments, which may not reflect the reality as observed by others.[6-8] Recently, Bernardo et al.,[8] showed that self-assessed empathy does not correlate with patient's measurements, strengthening the hypothesis that self-assessment may not be a reliable indicator of empathy change during the medical course.

Giving voice to patients may help health care providers to understand, acknowledge, and address patients' needs when working on a shared therapeutic plan.[9 10]. The success of a therapeutic plan depends on the competence of health professionals to understand and anticipate the challenges faced by their patients.[11-15] This competence relies on the empathetic attitudes of health professionals; attitudes that may open the door for meaningful conversations, allowing doctors to realize how patients' emotions and beliefs may interfere in the therapeutic process.[11 13] To deliver such competent doctors to society, medical schools need to develop curricular strategies to support medical students and residents along their journey towards empathy development as a strategy to consolidate patient-centered practices.[1 13 16]

Despite the many pedagogical interventions developed to foster empathy in medical students and residents, the medical education community is still concerned that medical schools are not yet

managing to nurture a culture that embraces empathy as a central aspect of medical professionalism.[16] The difficulty of understanding whether medical schools are fostering empathy may relate to the fact that most studies rely on self-assessment instruments for both longitudinal empathy measurement and assessment of empathy training' efficacy.[5] However, research shows that self-assessment may not be appropriate to measure behaviors and attitudes.[8 17] Thus, our question is to what extent self-assessed empathy should be the only parameter to explore how the medical culture and training affect the empathy levels of medical students and residents.

In the current article, we sought out to investigate the correlation between medical students' and residents' self-assessed empathy levels with the empathy levels as perceived by the patients assisted by them directly in real clinical encounters. We also investigated whether patients' measures are valid and reliable tools to be used as assessment methods of the empathy levels of medical students and residents. We believe that understanding how patients perceive the empathy of medical students and residents in the context of real medical encounters can support educational interventions to foster a more humanistic practice. Our research may help to enlighten the utilization of empathy measurements to guide educational practices.

METHODS

Context

We performed this research in three teaching public hospitals in São Paulo, Brazil. In all hospitals, interns and residents are independently responsible for the clinical consultations, which consists of two steps. First, the students or the residents interview and perform the clinical examination of the patient individually. Then, they leave the room to discuss the case with a supervisor to establish the principal diagnostic hypothesis, the differential diagnosis, and the treatment plan. Subsequently, they return to the room and share the plan with the patient. Thus, patients do not

For peer review only

interact directly with the supervisors, only with the trainees. The autonomy of interns and residents in this context creates the unique opportunity to study patients' perspectives on interns' and residents' empathy without the influence of patients having direct contact with the supervisor.

Participants

After each consultation in one of the hospital outpatient clinics, a researcher (MOB, ARAL, JFS, or HDC) invited patients to participate in the study. The researchers had no responsibility for patient care. The inclusion criteria were patients to be older than 18 years old, literate, and capable of filling the assessment instrument. In total, we invited 668 patients to participate in the study. We also invited to participate physicians in training who were either year five or year six senior medical students (interns) or year one to year three residents from diverse specialties. In total, we invited 112 physicians in training (interns + residents) to participate in the study.

Instruments

We used four instruments to measure empathy: two based on self-assessment and two based on direct observation by actual patients.

Self-assessment instruments

We used the Physician and Student Portuguese versions of the Jefferson Scale of Empathy (JSE),[18] and the Interpersonal Reactivity Index (IRI).[19] Both instruments have a mixture of positive and negative items inviting respondents to rate the extent to which they agree or disagree with each statement.

The Jefferson Scale of Physician Empathy (JSE) consists of 20 items rated on a 7-point Likert scale. JSE measures three subdimensions of empathy, "Perspective Taking," "Compassionate Care" and "Standing on the Patients' Shoes".[18 20] All the negative items were transformed into

For peer review only - http://bmjopen.bmj.com/site/about/guidelines.xhtml

positive to calculate the scores. The overall score for the JSE is the sum of all items' scores, and the scores of the subdimensions are the sum of their respective items.

The Interpersonal Reactivity Index (IRI*)* consists of 28 items rated on a 5-point Likert scale. IRI measures four subdimensions of empathy: "Perspective Taking," "Empathic Concern," "Personal Distress," and "Fantasy".[7 19] All negative items were transformed into positive to calculate the scores. The overall score for the IRI is the sum of all items scores, and the scores of the subdimensions are the sum of their respective items.

Patient's assessment instruments

To measure physicians' empathy as perceived by their patients, we used the Jefferson Scale of Patient's Perceptions of Physician Empathy (JSPPPE) and the Consultation and Relational Empathy scale (CARE).

The Jefferson Scale of Patient's Perceptions of Physician Empathy (JSPPPE) has 5-items rated on a 7-point Likert scale.[21 22] The overall score for the JSPPPE is the sum of all items' scores. We applied the validated Portuguese version.[8]

The Consultation and Relational Empathy Scale (CARE) instrument has ten items rated on a 6-point Likert scale.[23 24] The overall score for the CARE is the sum of all items scores. We used the validated Portuguese version of CARE.[18]

Study procedure

The sample of physicians in training is a convenient sample since the authors MOB, ARAL, JFS, and HDC had to seize the opportunity of inviting participants during their working hours in the referred university hospitals. The patient sample, on the contrary, comprehended all patients consulted by their respective physicians in training in the day of selection.

For peer review only

Patients were informed about the aim of the research and assured that participating in the study would not affect their care. Then, patients were invited to sign the consent form. Subsequently, only patients who signed the inform consent filled the questionnaires.

Physicians in training were informed about the aim of the research and that participating in the study would not affect their assessment during the clinical rotation. Then, physicians in training were asked to sign the inform consent. Only physicians in training who signed the inform consent filled the questionnaires.

We used paper questionnaires for both patients and physician in training. All forms were anonymized and inserted into a data system by a designated person, who did not have access to patients' names.

Data analyses

We compared patients' assessments considering their gender, physician's gender and physicians' training level (intern vs. resident) using t-tests. We also compared physicians' in training self-assessment of empathy in respect of their training level (intern vs. resident) using t-tests.

We used Pearson correlation to investigate the relation between physicians' in training self-assessed empathy with the empathy perceived by their patients. As the number of patients per physician in training differed (ranging from 3 to 15), we averaged the patients' responses to each physician in training before conducting the Pearson correlation analysis.

To investigate whether CARE and JSSSPE were valid and reliable tools for assessing physicians in training empathy, we conducted a confirmatory factor analysis with Maximum Likelihood estimation. We calculated the reliability using Cronbach alpha.

Ethical approval and procedures

We obtained ethical approval for this study from the Research Ethics Committee of the three

For peer review only

universities involved (university A - CAAE = 63847016.9.1001.5373; university B - CAAE = 63847016.9.2002.5404; and university C - CAAE = 63847016.9.2001.5415). All participants gave written informed consent before data collection.

RESULTS

From the 668 patients invited to participate in this research, 60 patients declined, and we excluded 40 protocols because patients were not able to complete the instruments. In total, 566 (84,7%) patients participated in this research, from three different university hospitals: university hospital A (n=237), university hospital B (n =151), and university hospital C (n=178). Of those, 238 (42%) were male, and 328 (58%) were female patients. The patients' age ranged from 18 to 77, with a mean of 47 years-old.

From the invited 112 physicians in training, 20 refused to participate, and we excluded six interns because of uncorrected filling. In total, 86 (76.8%) physicians in training from three university medical hospitals in Brazil (university hospital A – n= 36; university hospital B – n= 17, university hospital C – n=33) participated in this study. Of the 86 physicians in training, 35 (41%) were interns, and 51 (59%) were residents from clinical and surgical specialties. The gender distribution was 39 (45%) males and 47 (51%) females, with ages ranging from 22 to 33 years old. The residents' specialties were: surgery (n=21), internal medicine (n=21) and gynecology (n=9).

Patient assessments

Interns scored significantly higher than residents on both empathy patients' scales. Female physicians in training scored significantly higher on the JSPPE scale but not on the CARE scale. There were no differences in empathy scores according to patients' gender (Table 1).

For peer review only

Table 1. Descriptive and comparative statistics for empathy measurements by patients.

		n	JSPPPE	*P*	CARE	*P*
Physicians in training	Intern	191	33.27	P < 0.001	46.37	P < 0.001
	Resident	375	29.81		41.81	
Physicians' Gender	Male	276	30.04	P < 0.001	43.04	p > 0.05
	Female	290	31.87		43.63	
Patients' Gender	Male	238	31.31	p > 0.05	43.23	p > 0.05
	Female	328	30.73		43.43	

Abbreviations: JSPPE = the Jefferson Scale of Patient's Perceptions of Physician Empathy (JSPPPE); CARE = Consultation and Relational Empathy scale.

Physicians in training self-assessments

Interns scored higher than residents in both JSE and IRI scales, but we only found a significant difference for the JSE. Female physicians scored significantly higher in both JSE and IRI (Table 2).

Table 2. Descriptive and comparative statistics for empathy self-assessment by physicians in training.

		n	JSE	*P*	IRI	*P*
Physicians in training	Intern	35	121.14	**p < 0.05**	67.17	p > 0.05
	Resident	51	114.22		65.18	
Physicians' Gender	Male	39	112.90	**p < 0.01**	59.56	**p < 0.001**
	Female	47	120.47		71.32	

Abbreviations: JSE = the Jefferson Scale of Empathy (JSE); IRI = Interpersonal Reactivity Index.

For peer review only

Associations between patients' assessments and self-assessments of empathy

We did not find any correlation between the total scores of patients' and self-assessment scales. The same was true for the subdimensions of the scales, with one only exception. There was a positive and weak correlation of the JSPPPE score with the JSE Compassionate Care sub-score (Table 3).

Table 3. Pearson correlations between empathy measurements: self-assessments versus patients' assessments.

Physicians' perceptions (n = 86)		Patients' perceptions (n = 566)	
		JSPPPE	CARE
JSE	Perspective Taking	0.011	0.168
	Compassionate Care	0.241*	0.207
	Standing in the Patient´s Shoes	0.109	0.033
	Jefferson Total	0.149	0.196
IRI	Fantasy Scale	-0.013	0.172
	Perspective Taking	0.066	-0.067
	Empathic Concern	0.083	0.044
	Personal Distress	0.011	0.047
	IRI Total	0.046	0.089

Abbreviations: JSPPE = the Jefferson Scale of Patient's Perceptions of Physician Empathy (JSPPPE); CARE = Consultation and Relational Empathy scale; JSE = the Jefferson Scale of Empathy (JSE); IRI = Interpersonal Reactivity Index.

The validity and reliability of JSPPPE and CARE

The base model of confirmatory factor analysis for the JSPPPE scale (model A) displayed a moderate fit index values, based on the Tucker-Lewis Index (TLI), Comparative Fit Index (CFI) and Root Mean Square Error of Approximation (RMSEA). In model B, after we added the correlation between the items' errors, the model reached a satisfactory level of model fit (Table 4), demonstrating evidence of validity for the JSPPPE. Cronbach's alpha was 0.91, indicating that the instrument is reliable.

The base model of confirmatory factor analysis for the CARE scale (model A) displayed a moderate fit index values, based on the Tucker-Lewis Index (TLI), Comparative Fit Index (CFI) and Root Mean Square Error of Approximation (RMSEA). In model B, after we added the correlation between the items' errors, the model reached a satisfactory level of model fit (Table 4), demonstrating evidence of validity for the CARE. Cronbach's alpha was 0.96, indicating that the instrument is reliable.

Table 4. Fit index for the JSPPPE and CARE

		χ^2(df) Sig.	TLI	CFI	RMSEA (HI90)
JSPPPE	Model A	$\chi^2(5) = 30.177$; p<0.001	0.975	0.987	0.094 (0.128)
	Model B	$\chi^2(4) = 15.501$; p=0.004	0.986	0.994	0.071 (0.110)
CARE	Model A	$\chi^2(35) = 204.716$; p<0.001	0.960	0.969	0.093 (0.105)
	Model B	$\chi^2(26) = 51.538$; p=0.002	0.992	0.995	0.042 (0.058)

Abbreviations: JSPPE = the Jefferson Scale of Patient's Perceptions of Physician Empathy (JSPPPE); CARE = Consultation and Relational Empathy scale.

DISCUSSION

In this study, we sought to investigate whether empathy self-assessment by physicians in training correlated with their patients' perspectives. As a corollary, we also evaluated the validity and reliability of two instruments for patients' assessments of physicians in training.

Empathy is a complex construct that needs different lenses to be fully understood.[1 25] As expected, our results demonstrated that self-assessment of empathy by interns and residents does not correlate with patients' assessments, in alignment with the results previously observed for senior clinical practitioners.[17] This finding corroborates the general literature that points out the inaccuracy of self-assessment.[6 26] The observed mismatch raises the question of whether educational interventions to foster empathy should rely solely or preferably on self-assessment measurements to attest their quality or relevance. Although self-assessment may function as a stimulus to create awareness and motivation to change in trainees, patients' perspectives are crucial to improve the actual care and to verify the efficiency of pedagogical interventions.[8]

Our results also suggest that CARE and JSPPPE scales could be used as assessment tools and detect elements that may interfere in patients' perception of students and residents' empathy. Patients' assessments are a meaningful opportunity to engage trainees in a reflection on the relevance of developing themselves into empathic caregivers.[27 28] Inviting patients to share their perspectives allows physicians in training to gather feedback from the people they intend to care for; the people they should strive to understand, reassure and advise.[29] Furthermore, empowering patients as formal assessors reinforces the message that a good doctor acknowledges, reflects on, and reacts to patients' opinions and views.

Patients found interns more empathic than residents, which raises a concern about the effects of the transition to practice on physicians in training empathy levels. Although our study had a cross-

For peer review only

sectional design, it corroborates the importance of including patients' perspectives into this debate. Without giving voice to patients, we will not have a comprehensive understanding of how medical training affects empathy development of students and residents. Without patients' insights, we will also struggle to realize whether our pedagogical interventions are impacting students the way we have planned.

A limitation of the present study may lie in the fact that we did not explore additional elements that could influence patients' perceptions. For example, the time spent in the consultation, the time waiting for the consultation or the comfort of the environment may influence patients' measures.[30 31] Although these elements may have influenced patients' perspectives, the three hospitals where we collected the data are very similar. Another possible limitation is the limited number of physicians in training. However, the number of patients was high. This high number of patients allowed us (1) to decrease the bias, which may occur when one patient may have a different perspective of the others, and (2) to obtain a reliable measurement of the level of empathy of the physician in training.

This study adds evidence to the complexity of measuring empathy. If our ultimate goal is to increase physicians' empathy towards the patient, the assessment methods applied to evaluate the empathy levels of medical students and residents should include patients' perspectives. Including patients' perspectives pave the way for educational interventions to impact the reality of practice, which is the ultimate goal of medical education.

Concluding, our study demonstrated a mismatch between physicians in training empathy self-assessment and their patients' assessments. This finding may have two implications: (1) patients' instruments may be measuring a different component of empathy, and (2) the self-assessment of empathy probably is not enough to foster more humanistic patient care.

For peer review only

REFERENCES

1. Hojat M. Empathy in patient care: antecedents, development, measurement, and outcomes. New York: Springer Verlag 2007.

2. Derksen F, Bensing J, Lagro-Janssen A. Effectiveness of empathy in general practice: a systematic review. *Br J Gen Pract* 2012;63:e76-84.

3. Thirioux B, Birault F, Jaafari N. Empathy is a protective factor of burnout in physicians: new neuro-phenomenological hypotheses regarding empathy and sympathy in care relationship. *Front Psychol* 2016;7:763.

4. Hojat M, Gonnella JS, Mangione S, et al. Empathy in medical students as related to academic performance, clinical competence and gender. *Med Educ* 2002;36:522-7.

5. Ferreira-Valente A, Monteiro JS, Barbosa RM, et al. Clarifying changes in student empathy throughout medical school: a scoping review. *Adv Health Sci Educ Theory Pract* 2017;22:1293-313.

6. Eva KW, Regehr G. Self-assessment in the health professions: a reformulation and research agenda. *Acad Med* 2005;80:S46-54.

7. Davis M. Measuring individual differences in empathy: evidence for a multidimensional approach. *J Personal Soc Psychol* 1983;44:113-26.

8. Bernardo MO, Cecílio-Fernandes D, Costa P, et al. Physicians' self-assessed empathy levels do not correlate with patients' assessments. *Plos One* 2018;13:e0198488.

9. Stewart M, Brown JB, Donner A, et al. The impact of patient-centered care on outcomes. *J Family Pract* 2000;49:796-804.

For peer review only

10. Hojat M, DeSantis J, Gonnella JS. Patient perceptions of clinician's empathy: measurement and psychometrics. *J Patient Exp* 2017;4:78-83.

11. Grisso T, Appelbaum PS. Assessing competence to consent to treatment: A guide for physicians and other health professionals. Oxford: Oxford University Press 1998.

12. Kim SS, Kaplowitz S, Johnston MV. The effects of physician empathy on patient satisfaction and compliance. *Eval Health Prof* 2004;27:237-51.

13. Veloski J, Hojat M. Measuring specific elements of professionalism: empathy, teamwork, and lifelong learning. In: Stern DT Ed. Measuring Medical Professionalism. Oxford, UK: Oxford University Press 2006:117-25.

14. Street RL, Makoul G, Arora NK, et al. How does communication heal? Pathways linking clinician-patient communication to health outcomes. *Patient Educ Couns* 2009;74:295-301.

15. Merriel SW, Salisbury C, Metcalfe C, et al. Depth of the patient-doctor relationship and content of general practice consultations: cross-sectional study. *Br J Gen Pract* 2015;65:545-51.

16. Pedersen R. Empathy development in medical education: a critical review. *Med Teach* 2010;32:593-600.

17. Grosseman S, Novack DH, Duke P, et al. Residents' and standardized patients' perspectives on empathy: issues of agreement. *Patient Educ Couns* 2014;96:22-8.

18. Paro HBMS, Daud-Gallotti RM, Tibério IC, et al. Brazilian version of the Jefferson Scale of Empathy: psychometric properties and factor analysis. *BMC Med Educ* 2012;12:73.

19. Limpo T, Alves R, Castro SL. Medir a empatia: adaptação portuguesa do Índice de Reactividade Interpessoal. *Lab Psicol* 2010;8:171-84.

For peer review only

20. Hojat M, Gonnella JS, Nasca TJ, et al. The Jefferson Scale of Physician Empathy: further psychometric data and differences by gender and specialty at item level. *Acad Med* 2002;77:S58-60.

21. Kane GC, Gotto JL, Mangione S, et al. Jefferson Scale of Patient's perceptions of physician empathy: preliminary psychometric data. *Croatian Med J* 2007;48:81-6.

22. Hojat M, Louis DZ, Maxwell K, et al. Patient perceptions of physician empathy, satisfaction with physician, interpersonal trust, and compliance. *Int J Med Educ* 2010;1:83-7.

23. Mercer SW, Reilly D. A qualitative study of patient's views on the consultation at the Glasgow Homoeopathic Hospital, an NHS integrative complementary and orthodox medical care unit. *Patient Educ Couns* 2004;53:13-8.

24. Mercer SW, Maxwell M, Heaney D, et al. The consultation and relational empathy (CARE) measure: development and preliminary validation and reliability of an empathy-based consultation process measure. *Family Pract* 2004;21:699-705.

25. Smith KE, Norman GJ, Decety J. The complexity of empathy during medical school training: evidence for positive changes. *Med Educ* 2017;51:1146-59.

26. Davis DA, Mazmanian PE, Fordis M, et al. Accuracy of physician self-assessment compared with observed measures of competence: a systematic review. *JAMA* 2006;296:1094-102.

27. Bokken L, Rethans JJ, van Heurn L, et al. Students' views on the use of real patients and simulated patients in undergraduate medical education. *Acad Med* 2009;84:958-63.

For peer review only

28. Bokken L, Rethans JJ, Scherpbier AJ, et al. Strengths and weaknesses of simulated and real patients in the teaching of skills to medical students: a review. *Simul Healthcare* 2008;3:161-9.

29. Wensing M, Vingerhoets E, Grol R. Feedback based on patient evaluations: a tool for quality improvement? *Patient Educ Couns* 2003;51:149-53.

30. Neumann M, Bensing J, Wirtz M, et al. The impact of financial incentives on physician empathy: a study from the perspective of patients with private and statutory health insurance. *Patient Educ Couns* 2011;84:208-16.

31. Borracci RA, Doval HC, Celano L, et al. Patients' perceptions of Argentine physicians' empathy based on the Jefferson scale of patient's perceptions of physician empathy: Psychometric data and demographic differences. *Educ Health* 2017;30:19.

For peer review only

ACKNOWLEDGMENTS

We thank all the patients, interns and residents that allowed us to collect the data that supports our study.

FUNDING/SUPPORT

This study was funded by the "Fundação de Amparo à Pesquisa do Estado de São Paulo-FAPESP" – grant number: 2016/11908-1 and by the "Conselho Nacional de Desenvolvimento Científico e Tecnológico – CNPq" – grant number: 202319/2017-2.

CONFLICTS OF INTERESTS

The authors declare any conflict of interests.

DISCLAIMER

None.

PREVIOUS PRESENTATIONS

None.

DATA AVAILABILITY STATEMENT

All data relevant to the study are included in the article or uploaded as supplementary information.

For peer review only

Reporting checklist for cross sectional study.

Based on the STROBE cross sectional guidelines.

Instructions to authors

Complete this checklist by entering the page numbers from your manuscript where readers will find each of the items listed below.

Your article may not currently address all the items on the checklist. Please modify your text to include the missing information. If you are certain that an item does not apply, please write "n/a" and provide a short explanation.

Upload your completed checklist as an extra file when you submit to a journal.

In your methods section, say that you used the STROBE cross sectional reporting guidelines, and cite them as:

von Elm E, Altman DG, Egger M, Pocock SJ, Gotzsche PC, Vandenbroucke JP. The Strengthening the Reporting of Observational Studies in Epidemiology (STROBE) Statement: guidelines for reporting observational studies.

For peer review only

		Reporting Item	Page Number
Title	#1a	Indicate the study’s design with a commonly used term in the title or the abstract	1
Abstract	#1b	Provide in the abstract an informative and balanced summary of what was done and what was found	4
Background / rationale	#2	Explain the scientific background and rationale for the investigation being reported	6
Objectives	#3	State specific objectives, including any prespecified hypotheses	7
Study design	#4	Present key elements of study design early in the paper	7
Setting	#5	Describe the setting, locations, and relevant dates, including periods of recruitment, exposure, follow-up, and data collection	8
Eligibility criteria	#6a	Give the eligibility criteria, and the sources and methods of selection of participants.	8
	#7	Clearly define all outcomes, exposures, predictors, potential	8

		confounders, and effect modifiers. Give diagnostic criteria, if applicable	
Data sources / measurement	#8	For each variable of interest give sources of data and details of methods of assessment (measurement). Describe comparability of assessment methods if there is more than one group. Give information separately for for exposed and unexposed groups if applicable.	8-9
Bias	#9	Describe any efforts to address potential sources of bias	9
Study size	#10	Explain how the study size was arrived at	7
Quantitative variables	#11	Explain how quantitative variables were handled in the analyses. If applicable, describe which groupings were chosen, and why	10
Statistical methods	#12a	Describe all statistical methods, including those used to control for confounding	10
	#12b	Describe any methods used to examine subgroups and interactions	10
	#12c	Explain how missing data were addressed	10
	#12d	If applicable, describe analytical methods taking account of sampling strategy	10
	#12e	Describe any sensitivity analyses	10
Participants	#13a	Report numbers of individuals at each stage of study—eg numbers potentially eligible, examined for eligibility, confirmed eligible, included in the study, completing follow-up, and analysed. Give information separately for for exposed and unexposed groups if applicable.	7
	#13b	Give reasons for non-participation at each stage	7
	#13c	Consider use of a flow diagram	n/a
Descriptive data	#14a	Give characteristics of study participants (eg demographic, clinical, social) and information on exposures and potential confounders. Give information separately for exposed and unexposed groups if applicable.	11
	#14b	Indicate number of participants with missing data for each variable of interest	7
Outcome data	#15	Report numbers of outcome events or summary measures. Give information separately for exposed and unexposed groups if applicable.	n/a
Main results	#16a	Give unadjusted estimates and, if applicable, confounder-adjusted	n/a

For peer review only

		estimates and their precision (eg, 95% confidence interval). Make clear which confounders were adjusted for and why they were included	
	#16b	Report category boundaries when continuous variables were categorized	11-12
	#16c	If relevant, consider translating estimates of relative risk into absolute risk for a meaningful time period	n/a
Other analyses	#17	Report other analyses done—e.g., analyses of subgroups and interactions, and sensitivity analyses	11-12
Key results	#18	Summarise key results with reference to study objectives	13
Limitations	#19	Discuss limitations of the study, taking into account sources of potential bias or imprecision. Discuss both direction and magnitude of any potential bias.	14
Interpretation	#20	Give a cautious overall interpretation considering objectives, limitations, multiplicity of analyses, results from similar studies, and other relevant evidence.	13-14
Generalisability	#21	Discuss the generalisability (external validity) of the study results	14
Funding	#22	Give the source of funding and the role of the funders for the present study and, if applicable, for the original study on which the present article is based	18

The STROBE checklist is distributed under the terms of the Creative Commons Attribution License CC-BY. This checklist was completed on 22. January 2019 using https://www.goodreports.org/, a tool made by the EQUATOR Network in collaboration with Penelope.ai

For peer review only

BMJ Open

Investigating the relation between self-assessment and patients` assessments of physicians in training empathy: a multicentric observational cross-sectional study in three teaching hospitals in Brazil

Journal:	*BMJ Open*
Manuscript ID	bmjopen-2019-029356.R1
Article Type:	Research
Date Submitted by the Author:	15-Apr-2019

Complete List of Authors:	Bernardo, Mônica; Pontificia Universidade Catolica de Sao Paulo Faculdade de Ciencias Medicas e da Saude, Radiology<br>Cecilio-Fernandes, Dario; University Medical Center Groningen, CEDAR - Center for Education Development and Research in Health Professions<br>Lima, Alba; Medical Faculty of São Jose do Rio Preto<br>Silva, Julian; Universidade Estadual de Campinas Faculdade de Ciencias Medicas, Internal Medicine<br>Ceccato, Hugo; Universidade Estadual de Campinas Faculdade de Ciencias Medicas, Internal Medicine<br>Costa, Manuel; School of Health Sciences, University of Minho, Life and Health Sciences Research Institute<br>de Carvalho-Filho, Marco Antonio; University Medical Center Groningen, CEDAR - Center for Educational Development and Research in Health Sciences; Universidade Estadual de Campinas Faculdade de Ciencias Medicas, Internal Medicine

**Primary Subject Heading**:	Medical education and training
Secondary Subject Heading:	Communication, Patient-centred medicine
Keywords:	Empathy, MEDICAL EDUCATION & TRAINING, Internship, Residency

SCHOLARONE™
Manuscripts

Title: Investigating the relation between self-assessment and patients` assessments of physicians in training empathy: a multicentric observational cross-sectional study in three teaching hospitals in Brazil.

Mônica O. Bernardo, MD*, Dario Cecilio-Fernandes*, PhD, Alba R. A. Lima, PhD, Julian F. Silva, Hugo D. Ceccato, Manuel J. Costa, PhD, Marco A. Carvalho Filho, MD, PhD.

*These authors contributed equally to this work.

Authors' contributions:

MOB, MJC and MACF have substantially contributed to the design of the work. MOB, ARAL, JFS and HDC were responsible for the acquisition of data. DCF was responsible for the data analyses. MOB, DCF, MJC and MACF were responsible for the interpretation of the data. MOB and DCF were responsible for the first draft of the paper. All the authors have critically revised and approved the final version of the paper. All authors are accountable for all the aspects of the work.

Mônica Oliveira Bernardo* is PhD candidate in Medical Education, School of Medical Sciences, University of Campinas, Campinas, São Paul, Brazil, and assistant professor of medicine, Radiology Department, Faculty of Medicine, Catholic University of São Paulo, Sorocaba, São Paulo, Brazil.

For peer review only

Dario Cecilio-Fernandes* is Researcher at the Center for Education Development and Research in Health Professions (CEDAR), research group LEARN, the University of Groningen, University Medical Center Groningen; , and School of Medical Sciences, University of Campinas, Campinas, São Paulo, Brazil; ORCID: http://orcid.org/0000-0002-8746-1680

Alba Regina de Abreu Lima is professor of medicine, Medical Faculty of São Jose do Rio Preto, São Jose do Rio Preto, São Paulo, Brazil.

Julian Furtado Silva is a senior medical student, School of Medical Sciences, University of Campinas, Campinas, São Paulo, Brazil.

Hugo Dugolin Ceccato, is a senior medical student, School of Medical Sciences, University of Campinas, Campinas, São Paulo, Brazil.

Manuel João Costa is Associate Professor researcher at the Life and Health Sciences Research Institute, School of Medicine, University of Minho, Braga, Portugal; ORCID: https://orcid.org/0000-0001-5255-4257.

Marco Antonio de Carvalho-Filho is professor of medicine, undergraduate and postgraduate courses, Emergency Department, School of Medical Sciences, University of Campinas, Campinas, São Paulo, Brazil, Center for Educational Development Innovation and Research, University Medical Center, University of Groningen, Groningen; ORCID: http://orcid.org/0000-0001-7008-4092

For peer review only

Correspondence should be addressed to Marco Antonio de Carvalho-Filho, Onderwijsinstituut (OWI), UMCG, A Deusinglaan 1 (FC40), Building 3219, 9713 AV Groningen, The Netherlands, +31621330898 macarvalhofilho@gmail.com, m.a.de.carvalho.filho@umcg.nl.

Number all words: 2.522

For peer review only

ABSTRACT

Objectives: This study investigated the associations between self-assessed empathy levels by physicians in training and empathy levels as perceived by their patients after clinical encounters. The authors also examined whether patient assessments were valid and reliable tools to measure empathy in physicians in training.

Design: A multicentric observational cross-sectional study.

Setting: This study was conducted in three public teaching hospitals in Brazil.

Participants: From the 668 patients invited to participate in this research, 566 (84,7%) agreed. Of those, 238 (42%) were male, and 328 (58%) female patients. From the invited 112 physicians in training, 86 (76.8%) agreed. Of the 86 physicians in training, 35 (41%) were final years medical students and 51 (59%) were residents from clinical and surgical specialties. The gender distribution was 39 (45%) males and 47 (51%) females.

Primary and secondary outcome measures: Physicians in training filled the Jefferson Scale of Physician Empathy (JSE) and the Interpersonal Reactivity Index (IRI). The patients answered the Jefferson Scale of Patient's Perceptions of Physician Empathy (JSPPPE) and the Consultation and Relational Empathy Scale (CARE).

Results: This study found non-significant correlations between patient and physicians in training self-assessments, except for a weak correlation (0,241, $p<0.01$) between the JSPPPE score and the JSE Compassionate Care sub-score. CARE and JSPPPE scales proved to be valid and reliable instruments.

Conclusions: Physicians in training self-assessments of empathy differ from patient assessments. Knowledge about empathy derived from self-assessment studies probably does not capture the perspective of the patients, who are key stakeholders in patient centered care. Future research on

For peer review only - http://bmjopen.bmj.com/site/about/guidelines.xhtml

the development of physician empathy or on outcomes of educational interventions to foster empathy should include patient perspectives.

Strengths and limitations of this study:

- This was a multicentric study involving three public teaching hospitals.
- This study combined multiple perspectives of physicians in training empathy.
- The ratio of patients to physicians in training was high, thus decreasing the bias in patient assessments, resulting in reliable empathy measurements.
- This study did not take into account elements that may interfere with patients' experiences like the time spent in the consultation and/or waiting room.

Key words: Empathy. Education, Medical. Internship and Residency. Patients.

For peer review only

INTRODUCTION

Physician empathy is crucial for patient care.[1 2] Empathy enhances professional fulfillment,[1] diminishes physicians' burnout,[3] and is related to clinical competence.[4] Empathy is vital to understand, acknowledge, and address patients' needs in clinical encounters and to construct a shared and feasible therapeutic plan considering patients' beliefs and context.[5 6] Empathetic behaviour of health professionals is a cornerstone to establish meaningful conversations with patients, decrease anxiety and reveal patients' emotions and beliefs relevant to patients' experiences.[7-9]

The relevance of empathy to patient care has led to increasing calls to develop learning strategies to foster the capacity of physicians in training for empathic understanding.[4] Unfortunately, research findings with undergraduate students have raised concerns over eventual negative impacts of medical schools on student empathy (for a discussion see Ferreira-Valente et al.[10]). Despite the controversy over the effect of medical school on empathy, it is consensual that an important limitation of empathy research has been the frequent reliance on participant self-assessments, in general using the Jefferson Scale of Physician Empathy (JSE)[11] or the Interpersonal Reactivity Index (IRI)[12]. This is the case, for example, of longitudinal studies of medical student empathy[13] or of studies on outcomes of interventions to develop empathy.[14-16]

Despite the international use of established questionnaires, it is unknown to what extent empathy self-assessment scores reflect empathic behaviours as observed by others, specially patients.[17-19] The wider literature on self-assessments suggest these may be insufficient to capture the full breath of behaviors or attitudes.[19 20] It is therefore of paramount importance to characterize associations between empathy assessed by oneself and by others.

Recently, a multi-centre study in Brazil[19] of associations between self-reported and patient derived

empathy measures for physicians, reported that those may be unrelated, suggesting that doctor empathy self-assessments were not indicative of empathy, as perceived by patients. It is thus likely that the same is the case for resident or student empathy, but this remains to be confirmed empirically. Moreover, a recent study showed a lack of correlation between self-assessed empathy by primary care physicians and clinical outcomes in patients with Diabetes[21]. The lack of correlation between self-assessed empathy and both patients' assessments and clinical outcomes are powerful arguments to expand the field towards including patients' perspectives.

In the current article, we sought out to investigate the correlation between medical students' and residents' self-assessed empathy levels with the empathy levels as perceived by the patients assisted by them directly in real clinical encounters. We also investigated whether patients' measures are valid and reliable tools to be used as assessment methods of the empathy levels of medical students and residents. We believe that understanding how patients perceive the empathy of medical students and residents in the context of real medical encounters can inform educational interventions to foster a more humanistic practice. Our research may help to enlighten the utilization of empathy measurements to guide educational practices.

METHODS

Context

We performed this research in three teaching public hospitals in São Paulo, Brazil, in which interns and residents are independently responsible for the clinical consultations. There are two steps in any consultation. First, interns or residents interview and perform an autonomous clinical examination of the patient. Then, they meet the supervisor in another room to establish the principal diagnostic hypothesis, the differential diagnosis, and the treatment plan. Finally, they come back to their patients to share the plan. Thus, patients interact directly and exclusively with

For peer review only

the physicians in training (interns and residents), not with the supervisors. The autonomy of interns and residents in this context creates a unique opportunity to obtain patients' perspectives on interns' and residents' empathy, with no influence of supervisors.

Participants

Promptly after each consultation, a researcher (MOB, ARAL, JFS, or HDC) invited patients to participate in the study. The inclusion criteria were patients to be older than 18 years old, literate, and capable of filling the assessment instrument. In total, we invited 668 patients to participate in the study. None of the researchers had care responsibilities for any of the patients.

We invited to participate physicians in training who were either year five or year six senior medical students (interns) or year one to year three residents from diverse specialties. In total, we invited 112 physicians in training (interns + residents) to participate in the study.

Instruments

We used four instruments to measure empathy: two based on self-assessment and two based on direct observation by actual patients.

Self-assessment instruments

We used the Physician version of the Jefferson Scale of Empathy (JSE),[11] and the Interpersonal Reactivity Index (IRI).[12] These are the two scales most extensively used in empathy research. JSE was developed specifically for healthcare contexts whereas IRI was developed for the general population. Both instruments have a mixture of positive and negative items inviting respondents to rate the extent to which they agree or disagree with each statement.

The Jefferson Scale of Physician Empathy (JSE)[11] consists of 20 items rated on a 7-point Likert scale. JSE measures three subdimensions of empathy, "Perspective Taking," "Compassionate Care" and "Standing on the Patients' Shoes".[11] All the negative items were transformed into

For peer review only

positive to calculate the scores. The overall score for the JSE is the sum of all items' scores, and the scores of the subdimensions are the sum of their respective items.

The Interpersonal Reactivity Index (IRI*)* consists of 28 items rated on a 5-point Likert scale. IRI measures four subdimensions of empathy: "Perspective Taking," "Empathic Concern," "Personal Distress," and "Fantasy".[12] All negative items were transformed into positive to calculate the scores. The overall score for the IRI is the sum of all items scores, and the scores of the subdimensions are the sum of their respective items.

We used the Physician and Student Portuguese versions of the Jefferson Scale of Empathy (JSE),[22] and Interpersonal Reactivity Index (IRI).[23]

Patient's assessment instruments

To measure physicians' empathy as perceived by their patients, we used the Jefferson Scale of Patient's Perceptions of Physician Empathy (JSPPPE)[24] and the Consultation and Relational Empathy scale (CARE).[25]

We used JSPPPE because it shares the same concept of empathy as the JSE – both were developed by the same research group.[24] CARE was developed aiming the concept of empathy as perceived by patients and there is evidence of the reliability, internal validity and consistency of this scale.[26]

The Jefferson Scale of Patient's Perceptions of Physician Empathy (JSPPPE) has 5-items rated on a 7-point Likert scale.[24 27] The overall score for the JSPPPE is the sum of all items' scores. We applied the validated Portuguese version.[19]

The Consultation and Relational Empathy Scale (CARE) instrument has ten items rated on a 6-point Likert scale.[25-26] The overall score for the CARE is the sum of all items scores. We used the validated Portuguese version of CARE.[28]

For peer review only - http://bmjopen.bmj.com/site/about/guidelines.xhtml

Previous studies had demonstrated that JSPPPE and CARE were unidimensional with high reliability coefficients – respectively 0.88 and 0.97[19] As the Portuguese version of these instruments had only been validated with a sample of medical doctors in Brazil, we have conducted new analysis to investigate whether the psychometric properties were similar for physicians in training.

Study procedure

The sample of physicians in training is a convenient sample since the authors MOB, ARAL, JFS, and HDC had to seize the opportunity of inviting participants during their working hours in the referred university hospitals. The patient sample, on the contrary, comprehended all patients consulted by their respective physicians in training in the day of selection.

Patients were informed about the aim of the research and assured that participating in the study would not affect their care. Then, patients were invited to sign the consent form. Subsequently, only patients who signed the inform consent filled the questionnaires.

Physicians in training were informed about the aim of the research and that participating in the study would not affect their assessment during the clinical rotation. Then, physicians in training were asked to sign the inform consent. Only physicians in training who signed the inform consent filled the questionnaires. The physicians in training filled the questionnaires only once before we started collecting the patients' questionnaires.

We used paper questionnaires for both patients and physician in training. All forms were anonymized and inserted into a data system by a designated person, who did not have access to patients' names.

Data analyses

For peer review only

We compared patients' assessments considering their gender, physician's gender and physicians' training level (intern vs. resident) using t-tests. We also compared physicians' in training self-assessment of empathy in respect of their training level (intern vs. resident) using t-tests.

We used Pearson correlation to investigate the relation between physicians' in training self-assessed empathy with the empathy perceived by their patients. As the number of patients per physician in training differed (ranging from 3 to 15), we averaged the patients' responses to each physician in training before conducting the Pearson correlation analysis.

To investigate whether CARE and JSSSPE were valid and reliable tools in specific study population, we conducted a confirmatory factor analysis with Maximum Likelihood estimation. We calculated the reliability using Cronbach alpha. We also investigated the concurrent validity of the scales by comparing the scores of JSSPE and CARE using Pearson correlation.

Ethical approval and procedures

We obtained ethical approval for this study from the Research Ethics Committee of the three universities involved (university A - CAAE = 63847016.9.1001.5373; university B - CAAE = 63847016.9.2002.5404; and university C - CAAE = 63847016.9.2001.5415). All participants gave written informed consent before data collection.

Patient and Public Involvement

Our research sought out to explore the potential contribution of patients' feedback on physicians in training empathy aiming a better quality of patient care and experience. We involved patients who voluntarily accepted to participate in a random selection. Patients were not involved in study design. The results of our study will be available for all the institutions and their patient representatives.

For peer review only

RESULTS

From the 668 patients invited to participate in this research, 60 patients declined, and 40 were excluded due to difficulties in completing the instruments. In total, 566 (84,7%) patients participated in this research, from three different university hospitals: university hospital A (n=237), university hospital B (n =151), and university hospital C (n=178). Of those, 238 (42%) were male, and 328 (58%) were female patients. The patients' age ranged from 18 to 77, with a mean of 47 years-old.

From the invited 112 physicians in training, 20 refused to participate, and 6 were excluded because of uncorrected filling of JSE or IRI. In total, 86 (76.8%) physicians in training from three university medical hospitals in Brazil (university hospital A – n= 36; university hospital B – n= 17, university hospital C – n=33) participated in this study. Of the 86 physicians in training, 35 (41%) were interns, and 51 (59%) were residents from clinical and surgical specialties. The gender distribution was 39 (45%) males and 47 (51%) females, with ages ranging from 22 to 33 years old. The residents' specialties were: surgery (n=21), internal medicine (n=21) and gynecology (n=9).

Physicians in training self-assessments

Interns scored higher than residents in both JSE and IRI scales, but the differences were only statistically significant for the JSE. Female physicians scored significantly higher in both JSE and IRI (Table 1).

Table 1. Descriptive and comparative statistics for empathy self-assessment by physicians in training.

		n	JSE (SD)	*P*	IRI (SD)	*P*
Physicians in training	Intern	35	121.14 (9.52)	**$p < 0.05$**	67.17 (11.56)	$p > 0.05$

For peer review only

	Resident	51	114.22 (14.26)		65.18 (14.02)	
Physicians' Gender	Male	39	112.90 (14.6)	**p < 0.01**	59.56 (13.33)	**p < 0.001**
	Female	47	120.47 (10.35)		71.32 (10.18)	

Abbreviations: JSE = the Jefferson Scale of Empathy (JSE); IRI = Interpersonal Reactivity Index.

We found a moderate and significant correlation between JSE and IRI ($r=0.44$, $p < 0.05$). We also found positive and significant correlations between the sub-scales ranging from weak to moderate magnitude (Table 2)

Table 2. Pearson correlations between JSE and IRI.

		IRI				
		Fantasy Scale	Perspective Taking	Empathic Concern	Personal Distress	IRI Total
JSE	Perspective Taking	0.355*	0.285*	0.632*	-0.048	0.485*
	Compassionate Care	0.364*	0.342*	0.603*	0.046	0.346*
	Standing in the Patient´s Shoes	0.318*	0.184	0.492*	-0.035	0.031
	Jefferson Total	0.033	0.038	0.240*	-0.183	0.435*

Abbreviations: JSE = the Jefferson Scale of Empathy (JSE); IRI = Interpersonal Reactivity Index.

Patient assessments

Interns scored significantly higher than residents on both empathy patients' scales. Female physicians in training scored significantly higher on the JSPPE scale but not on the CARE scale. There were no differences in empathy scores according to patients' gender (Table 3).

For peer review only

Table 3. Descriptive and comparative statistics for empathy measurements by patients.

		n	JSPPPE (SD)	*P*	CARE (SD)	*P*
Physicians in training	Intern	191	33.27 (3.59)	P < 0.001	46.37 (6.95)	P < 0.001
	Resident	375	29.81 (6.68)		41.81 (8.09)	
Physicians' Gender	Male	276	30.04 (6.72)	P < 0.001	43.04 (8.25)	p > 0.05
	Female	290	31.87 (5.18)		43.63 (7.8)	
Patients' Gender	Male	238	31.31 (5.78)	p > 0.05	43.23 (8.3)	p > 0.05
	Female	328	30.73 (6.22)		43.43 (7.8)	

Abbreviations: JSPPE = the Jefferson Scale of Patient's Perceptions of Physician Empathy (JSPPPE); CARE = Consultation and Relational Empathy scale.

Associations between patients' assessments and self-assessments of empathy

We did not find any correlation between the total scores of patients' and self-assessment scales. The same was true for the subdimensions of the scales, with one only exception. There was a positive and weak correlation of the JSPPPE score with the JSE Compassionate Care sub-score (Table 4).

For peer review only

Table 4. Pearson correlations between empathy measurements: self-assessments versus patients' assessments.

Physicians' perceptions (n = 86)		Patients' perceptions (n = 566)	
		JSPPPE	CARE
JSE	Perspective Taking	0.011	0.168
	Compassionate Care	0.241*	0.207
	Standing in the Patient´s Shoes	0.109	0.033
	Jefferson Total	0.149	0.196
IRI	Fantasy Scale	-0.013	0.172
	Perspective Taking	0.066	-0.067
	Empathic Concern	0.083	0.044
	Personal Distress	0.011	0.047
	IRI Total	0.046	0.089

Abbreviations: JSPPE = the Jefferson Scale of Patient's Perceptions of Physician Empathy (JSPPPE); CARE = Consultation and Relational Empathy scale; JSE = the Jefferson Scale of Empathy (JSE); IRI = Interpersonal Reactivity Index.

Validity and reliability of JSPPPE and CARE

The base model of confirmatory factor analysis for the JSPPPE scale (model A) displayed a moderate fit index values, based on the Tucker-Lewis Index (TLI), Comparative Fit Index (CFI) and Root Mean Square Error of Approximation (RMSEA). In model B, after we added the correlation between the items' errors, the model reached a satisfactory level of model fit (Table 5), demonstrating evidence of validity for the JSPPPE. Cronbach's alpha was 0.91, indicating that the instrument is reliable.

For peer review only - http://bmjopen.bmj.com/site/about/guidelines.xhtml

The base model of confirmatory factor analysis for the CARE scale (model A) displayed a moderate fit index values, based on the Tucker-Lewis Index (TLI), Comparative Fit Index (CFI) and Root Mean Square Error of Approximation (RMSEA). In model B, after we added the correlation between the items' errors, the model reached a satisfactory level of model fit (Table 5), demonstrating evidence of validity for the CARE. Cronbach's alpha was 0.96, indicating that the instrument is reliable.

Table 5. Fit index for the JSPPPE and CARE

		χ^2(df) Sig.	TLI	CFI	RMSEA (HI90)
JSPPPE	Model A	$\chi^2(5) = 30.177$; p<0.001	0.975	0.987	0.094 (0.128)
	Model B	$\chi^2(4) = 15.501$; p=0.004	0.986	0.994	0.071 (0.110)
CARE	Model A	$\chi^2(35) = 204.716$; p<0.001	0.960	0.969	0.093 (0.105)
	Model B	$\chi^2(26) = 51.538$; p=0.002	0.992	0.995	0.042 (0.058)

Abbreviations: JSPPE = the Jefferson Scale of Patient's Perceptions of Physician Empathy (JSPPPE); CARE = Consultation and Relational Empathy scale.

DISCUSSION

In this study, we sought to investigate whether empathy self-assessment by physicians in training correlated with their patients' assessments. We also evaluated the validity and reliability of the two instruments for patients' assessments of physicians in training. Our findings corroborated the hypothesis that self-assessment of empathy by interns and residents did not correlate with patients' assessments, in line with findings with senior clinical practitioners.[19] Taking into consideration

For peer review only - http://bmjopen.bmj.com/site/about/guidelines.xhtml

that this study used the 4 empathy scales– 2 self and 2 patient assessments – most widely used on empathy research, the findings are of particular relevance. A former study in five countries had demonstrated that the two self-reported scales did not capture the same empathy construct.[29] The lack of correlation between self- and patient assessed empathy has implications for how the literature on health professional's empathy is interpreted. Quite likely, findings from studies using any of the 4 scales, which often compare empathy across studies, are not directly comparable. Like in the parabola of the elephant and the blind man in which each blind man feels a different piece of the elephant, it is possible that such studies capture different elements of the complex psychological construct called empathy. To develop empathy studies relevant to inform medical education, it is crucial, at this moment, to clarify which scale – if any – offers measure which correlates with meaningful clinical or educational outcomes. For example, recently, Chaitoff et al.[21] found that self-assessed empathy levels of primary-care physicians were not correlated with laboratorial outcomes in patients with Diabetes. This result enlightens the debate on the correlation between self-assessed empathy and clinical outcomes by showing that a relationship of cause-effect between those two variables is unlikely.

Empathy is a complex construct with cognitive, affective, behavioral and moral dimensions entailing different lenses to be fully understood.[1 30] When patients are invited to discuss what is a positive outcome through their perspectives, the complexity increases. Patients' definition of a positive outcome may vary along the course of their disease and life. We believe that empathy is a necessary psychological trait for the doctor to understand the singularity of each patient and individualize therapeutic plans in alignment with patients' needs and beliefs. Considering both the complexity of empathy and the singularity of patients' experiences, we invite researchers in this field to expand their focus.

For peer review only

First, longitudinal studies or pre/post-test evaluations of learning strategies using self-reported empathy cannot anticipate physicians in training performance on real clinical encounters. We agree that self-reported measurements can help teachers to start the conversation with students around the relevance of being empathic for becoming a caregiver. However, if educators want to mirror future performance, it is crucial to include real patients in the assessment of students. Specially during the transition to independent practice, when students face the challenges related to adapting to the constrains of the health care system, medical educators should reinforce the importance of empathy, while helping students to align theory and practice through effective role-modeling.[31]

Second, studies targeting the understanding of the possible association between empathy and clinical outcomes should take into consideration the importance of empathy on building a therapeutic alliance between the doctor and the patient. So, we hypothesize that punctual assessments by patients after a singular encounter are not enough to capture the phenomena under study and we should use instruments to measure the quality of the relationship between the doctor and patient. However, we do believe that punctual assessments may be efficient to give feedback to clinicians on their attitudes and behaviors nurturing the development of their communication skills.

As expected, our results demonstrated that self-assessment of empathy by interns and residents does not correlate with patients' assessments, in alignment with the results previously observed for senior clinical practitioners.[19] This finding corroborates the general literature that points out the inaccuracy of self-assessment.[17 18] Overall, physicians in training might become overconfident over time. For example, consecutive participation in clinical practice may increase students' self-confidence[32 33], which does not necessarily predict their performance. Furthermore, self-assessment questionnaires in empathy often focus only on whether the participants are aware of

For peer review only

how they have to behave to be empathic. However, knowing how to behave does not necessarily translates into a change of the behavior in practice. Our results are aligned with this possible mismatch between intention and action.

Our results also suggest that CARE and JSPPPE scales could be used as assessment tools and detect elements that may interfere in patients' perception of students and residents' empathy. More importantly, the psychometric properties of the instrument are very similar when looking at the physicians in training and medical doctors.[19] Both CARE and JSPPPE have followed the same internal structure and similar reliability coefficient. Contradictory to previous studies where weak correlations were found between JSE and IRI,[29] our results indicated a moderated correlation between the JSE and IRI, suggesting that both scales may be measuring the same aspect of empathy. Although this finding adds to the number of validity evidence of the JSE, the outcomes should be carefully interpreted since it seems that the relation may be related to the sample and context.

Patients found interns more empathic than residents, which raises a concern about the effects of the transition to practice on physicians in training empathy levels. Previous studies have shown a decline in self-assessed empathy levels during undergraduate medical training[34], but this finding is not universal across different medical schools[10]. Studies evaluating self-assessed empathy levels during residency training showed heterogeneous results.[35-37] Our study was not designed to investigate the evolution of empathy throughout the maturation of doctors, but it raises the possibility that empathy as perceived by patients can decrease during the transition from internship to residency training. Patients also considered female interns and residents to be more empathetic than males, a phenomenon that is also observed with self-assessed empathy.[38]

For peer review only

Although our study had a cross-sectional design, it corroborates the importance of including patients' perspectives into this debate. Without giving voice to patients, we will not have a comprehensive understanding of how medical training affects empathy development of students and residents. Without patients' insights, we will also struggle to realize whether our pedagogical interventions are impacting students the way we have planned.

A limitation of the present study is that we did not address the influence on patient perceptions of contextual or environmental factors, such as the consultation time, delay in the waiting room or the comfort of the environment. These elements may have influenced patients' perspectives.[39 40] Another limitation was the study's inability to pair in time the assessments of physicians and patients. Also, the sample of physicinans in training was not randomly selected. A final limitation was that participants were informed of the nature of the research, which may have induced behaviors more socially desirable and have biased the results.

The relatively large number of patients is one of the strengths of this study. Such high number of patients allowed us (1) to decrease the bias, which may occur when one patient may have a different perspective of the others, and (2) to obtain a reliable measurement of the level of empathy of the physician in training.

This study adds evidence to the complexity of measuring empathy. The observed mismatch raises the question of whether educational interventions to foster empathy should rely solely or preferably on self-assessment measurements to attest their quality or relevance. Although self-assessment may function as a stimulus to create awareness and motivation to change in trainees, patients' perspectives are crucial to improve the actual care and to verify the efficiency of pedagogical interventions.[19]

For peer review only

Patients' assessments are a meaningful opportunity to engage trainees in a reflection on the relevance of developing themselves into empathic caregivers.[41-42] Inviting patients to share their perspectives allows physicians in training to gather feedback from the people they intend to care for; the people they should strive to understand, reassure and advise.[43] Furthermore, empowering patients as formal assessors reinforces the message that a good doctor acknowledges, reflects on, and reacts to patients' opinions and views.

If our ultimate goal is to increase physicians' empathy towards the patient, the assessment methods applied to evaluate the empathy levels of medical students and residents should include patients' perspectives. Including patients' perspectives pave the way for educational interventions to impact the reality of practice, which is the ultimate goal of medical education.

Concluding, our study demonstrated a mismatch between physicians in training empathy self-assessment and their patients' assessments. This finding may have two implications: (1) patients' instruments may be measuring a different component of empathy, and (2) the self-assessment of empathy probably is not enough to foster more humanistic patient care.

REFERENCES

1. Hojat M. Empathy in patient care: antecedents, development, measurement, and outcomes. New York: Springer Verlag 2007.

2. Derksen F, Bensing J, Lagro-Janssen A. Effectiveness of empathy in general practice: a systematic review. Br J Gen Pract 2012;63:e76-84.

For peer review only

3. Thirioux B, Birault F, Jaafari N. Empathy is a protective factor of burnout in physicians: new neuro-phenomenological hypotheses regarding empathy and sympathy in care relationship. Front Psychol 2016;7:763.

4. Hojat M, Gonnella JS, Mangione S, et al. Empathy in medical students as related to academic performance, clinical competence and gender. Med Educ 2002;36:522-7.

5. Stewart M, Brown JB, Donner A, et al. The impact of patient-centered care on outcomes. J Family Pract 2000;49:796-804.

6. Hojat M, DeSantis J, Gonnella JS. Patient perceptions of clinician's empathy: measurement and psychometrics. J Patient Exp 2017;4:78-83.

7. Grisso T, Appelbaum PS. Assessing competence to consent to treatment: A guide for physicians and other health professionals. Oxford: Oxford University Press 1998.

8. Veloski J, Hojat M. Measuring specific elements of professionalism: empathy, teamwork, and lifelong learning. In: Stern DT Ed. Measuring Medical Professionalism. Oxford, UK: Oxford University Press 2006:117-25.

9. Weiss R, Vittinghoff E, Fang MC, et al. Associations of physician empathy with patient anxiety and ratings of communication in hospital admission encounters. J Hosp Med 2017;12:805-10.

10. Ferreira-Valente A, Monteiro JS, Barbosa RM, et al. Clarifying changes in student empathy throughout medical school: a scoping review. Adv Health Sci Educ Theory Pract 2017;22:1293-313.

For peer review only

11. Hojat M, Mangione S, Nasca TJ, Cohen MJM, Gonnella JS, Erdmann JB, et al. The Jefferson Scale of Physician Empathy: development and preliminary psychometric data. Educ Psychol Meas. 2001;61:349-65.

12. Davis M. Measuring individual differences in empathy: evidence for a multidimensional approach. J Personal Soc Psychol 1983;44:113-26.

13. Costa P, Magalhães E, Costa MJ. A latent growth model suggests that empathy of medical students does not decline over time. Adv Health Sci Educ 2013;18(3):509-22.

14. Schweller M, Costa FO, Antônio MA, et al. The impact of simulated medical consultations on the empathy levels of students at one medical school. Acad Med 2014;89(4):632-7.

15. Hojat M, Axelrod D, Spandorfer J, et al. Enhancing and sustaining empathy in medical students. Med Teach 2013;35:996-1001.

16. Batt-Rawden SA, Chisolm MS, Anton B, et al. Teaching empathy to medical students: an updated, systematic review. Acad Med 2013;88(8):1171-7.

17. Eva KW, Regehr G. Self-assessment in the health professions: a reformulation and research agenda. Acad Med 2005;80:S46-54.

18. Davis DA, Mazmanian PE, Fordis M, et al. Accuracy of physician self-assessment compared with observed measures of competence: a systematic review. JAMA 2006;296:1094-102.

For peer review only

19. Bernardo MO, Cecílio-Fernandes D, Costa P, et al. Physicians' self-assessed empathy levels do not correlate with patients' assessments. Plos One 2018;13:e0198488.

20. Grosseman S, Novack DH, Duke P, et al. Residents' and standardized patients' perspectives on empathy: issues of agreement. Patient Educ Couns 2014;96:22-8.

21. Chaitoff A, Rothberg MB, Windover AK, et al. Physician empathy is not associated with laboratory outcomes in diabetes: a cross-sectional study. J Gen Intern Med 2019;34(1):75-81.

22. Paro HBMS, Daud-Gallotti RM, Tibério IC, et al. Brazilian version of the Jefferson Scale of Empathy: psychometric properties and factor analysis. BMC Med Educ 2012;12:73.

23. Limpo T, Alves R, Castro SL. Medir a empatia: adaptação portuguesa do Índice de Reactividade Interpessoal. Lab Psicol 2010;8:171-84.

24. Kane GC, Gotto JL, Mangione S, et al. Jefferson Scale of Patient's perceptions of physician empathy: preliminary psychometric data. Croatian Med J 2007;48:81-6.

25. Mercer SW, Maxwell M, Heaney D, et al. The consultation and relational empathy (CARE) measure: development and preliminary validation and reliability of an empathy-based consultation process measure. Family Pract 2004;21:699-705.

26. Mercer SW, Reilly D. A qualitative study of patient's views on the consultation at the Glasgow Homoeopathic Hospital, an NHS integrative complementary and orthodox medical care unit. Patient Educ Couns 2004;53:13-8.

For peer review only

27. Hojat M, Louis DZ, Maxwell K, et al. Patient perceptions of physician empathy, satisfaction with physician, interpersonal trust, and compliance. Int J Med Educ 2010;1:83-7.

28. Scarpellini GR, Capellato G, Rizzatti FG, Silva GA, Baddini-Martinez JA. CARE Scale of Empaty: translation to the Portuguese spoken in Brazil and initial validation results. Medicina (São Paulo). 2014;47:51-8.

29. Costa P, Carvalho-Filho MA, Schweller M, et al. Measuring medical students' empathy: exploring the underlying constructs of and associations between two widely used self-report instruments in five countries. Acad Med 2017;92(6):860-7.

30. Smith KE, Norman GJ, Decety J. The complexity of empathy during medical school training: evidence for positive changes. Med Educ 2017;51:1146-59.

31. Laughey W, Sangvik Grandal N, Stockbridge C, et al. Twelve tips for teaching empathy using simulated patients. Med Teach 2018 Jun 26:1-5. [Epub ahead of print]

32. Cleave-Hogg D, Morgan PJ. Experiential learning in an anaesthesia simulation centre: analysis of students' comments. Med Teach 2002;24:23-26.

33. Dornan T, Scherpbier A, King N, et al. Clinical teachers and problem-based learning: a phenomenological study. Med Educ 2005;39(2):163-70.

34. Hojat M, Vergare MJ, Maxwell K, Brainard G, Herrine SK, Isenberg GA, et al. The devil is in the third year: A longitudinal study of erosion of empathy in medical school. Acad Med. 2009;84(9):1182-91.

For peer review only

35. Avasarala SK, Whitehouse S, Drake SM. Internship and empathy: variations across time and specialties. South Med J 2015;108(10):591-5.

36. Bratek A1, Bulska W, Bonk M, et al. Empathy among physicians, medical students and candidates. Psychiatr Danub. 2015;27 Suppl 1:S48-52.

37. Greenberg L, Agrawal D, Toto R, et al. Empathy, sense of power, and personality: do they change during pediatric residency? South Med J 2015;108(8):471-4.

38. Hojat M, Gonnella JS, Nasca TJ, Mangione S, Vergare M, Magee M. Physician empathy: definition, components, measurement, and relationship to gender and specialty. Am J Psychiatry. 2002;159(9):1563-9.

39. Neumann M, Bensing J, Wirtz M, et al. The impact of financial incentives on physician empathy: a study from the perspective of patients with private and statutory health insurance. Patient Educ Couns 2011;84:208-16.

40. Borracci RA, Doval HC, Celano L, et al. Patients' perceptions of Argentine physicians' empathy based on the Jefferson scale of patient's perceptions of physician empathy: Psychometric data and demographic differences. Educ Health 2017;30:19.

41. Bokken L, Rethans JJ, van Heurn L, et al. Students' views on the use of real patients and simulated patients in undergraduate medical education. Acad Med 2009;84:958-63.

42. Bokken L, Rethans JJ, Scherpbier AJ, et al. Strengths and weaknesses of simulated and real patients in the teaching of skills to medical students: a review. Simul Healthcare 2008;3:161-9.

For peer review only

43. Wensing M, Vingerhoets E, Grol R. Feedback based on patient evaluations: a tool for quality improvement? Patient Educ Couns 2003;51:149-53.

ACKNOWLEDGMENTS

We thank all the patients, patients' advisors, interns and residents that allowed us to collect the data that supports our study.

FUNDING/SUPPORT

This study was funded by the "Fundação de Amparo à Pesquisa do Estado de São Paulo-FAPESP" – grant number: 2016/11908-1 and by the "Conselho Nacional de Desenvolvimento Científico e Tecnológico – CNPq" – grant number: 202319/2017-2.

CONFLICTS OF INTERESTS

The authors declare no conflict of interests.

DISCLAIMER

None.

PREVIOUS PRESENTATIONS

None.

DATA AVAILABILITY STATEMENT

All data relevant to the study are included in the article or uploaded as supplementary information.

For peer review only

Reporting checklist for cross sectional study.

Based on the STROBE cross sectional guidelines.

Instructions to authors

Complete this checklist by entering the page numbers from your manuscript where readers will find each of the items listed below.

Your article may not currently address all the items on the checklist. Please modify your text to include the missing information. If you are certain that an item does not apply, please write "n/a" and provide a short explanation.

Upload your completed checklist as an extra file when you submit to a journal.

In your methods section, say that you used the STROBE cross sectional reporting guidelines, and cite them as:

von Elm E, Altman DG, Egger M, Pocock SJ, Gotzsche PC, Vandenbroucke JP. The Strengthening the Reporting of Observational Studies in Epidemiology (STROBE) Statement: guidelines for reporting observational studies.

		Reporting Item	Page Number
Title	#1a	Indicate the study's design with a commonly used term in the title or the abstract	1
Abstract	#1b	Provide in the abstract an informative and balanced summary of what was done and what was found	4
Background / rationale	#2	Explain the scientific background and rationale for the investigation being reported	6
Objectives	#3	State specific objectives, including any prespecified hypotheses	7
Study design	#4	Present key elements of study design early in the paper	7
Setting	#5	Describe the setting, locations, and relevant dates, including periods of recruitment, exposure, follow-up, and data collection	8
Eligibility criteria	#6a	Give the eligibility criteria, and the sources and methods of selection of participants.	8
	#7	Clearly define all outcomes, exposures, predictors, potential	8

For peer review only

		confounders, and effect modifiers. Give diagnostic criteria, if applicable	
Data sources / measurement	#8	For each variable of interest give sources of data and details of methods of assessment (measurement). Describe comparability of assessment methods if there is more than one group. Give information separately for for exposed and unexposed groups if applicable.	8-9
Bias	#9	Describe any efforts to address potential sources of bias	9
Study size	#10	Explain how the study size was arrived at	7
Quantitative variables	#11	Explain how quantitative variables were handled in the analyses. If applicable, describe which groupings were chosen, and why	10
Statistical methods	#12a	Describe all statistical methods, including those used to control for confounding	10
	#12b	Describe any methods used to examine subgroups and interactions	10
	#12c	Explain how missing data were addressed	10
	#12d	If applicable, describe analytical methods taking account of sampling strategy	10
	#12e	Describe any sensitivity analyses	10
Participants	#13a	Report numbers of individuals at each stage of study—eg numbers potentially eligible, examined for eligibility, confirmed eligible, included in the study, completing follow-up, and analysed. Give information separately for for exposed and unexposed groups if applicable.	7
	#13b	Give reasons for non-participation at each stage	7
	#13c	Consider use of a flow diagram	n/a
Descriptive data	#14a	Give characteristics of study participants (eg demographic, clinical, social) and information on exposures and potential confounders. Give information separately for exposed and unexposed groups if applicable.	11
	#14b	Indicate number of participants with missing data for each variable of interest	7
Outcome data	#15	Report numbers of outcome events or summary measures. Give information separately for exposed and unexposed groups if applicable.	n/a
Main results	#16a	Give unadjusted estimates and, if applicable, confounder-adjusted	n/a

For peer review only

		estimates and their precision (eg, 95% confidence interval). Make clear which confounders were adjusted for and why they were included	
	#16b	Report category boundaries when continuous variables were categorized	11-12
	#16c	If relevant, consider translating estimates of relative risk into absolute risk for a meaningful time period	n/a
Other analyses	#17	Report other analyses done—e.g., analyses of subgroups and interactions, and sensitivity analyses	11-12
Key results	#18	Summarise key results with reference to study objectives	13
Limitations	#19	Discuss limitations of the study, taking into account sources of potential bias or imprecision. Discuss both direction and magnitude of any potential bias.	14
Interpretation	#20	Give a cautious overall interpretation considering objectives, limitations, multiplicity of analyses, results from similar studies, and other relevant evidence.	13-14
Generalisability	#21	Discuss the generalisability (external validity) of the study results	14
Funding	#22	Give the source of funding and the role of the funders for the present study and, if applicable, for the original study on which the present article is based	18

The STROBE checklist is distributed under the terms of the Creative Commons Attribution License CC-BY. This checklist was completed on 22. January 2019 using https://www.goodreports.org/, a tool made by the EQUATOR Network in collaboration with Penelope.ai

For peer review only
